# Polarization insensitive split square ring resonator based epsilon-negative and near zero refractive index metamaterial for S, C, and X frequency bands satellite and radar communications

**DOI:** 10.1038/s41598-022-12322-1

**Published:** 2022-06-03

**Authors:** Ismail Hossain, Mohammad Tariqul Islam, Md. Samsuzzaman, Md. Moniruzzaman, Norsuzlin Binti Mohd Sahar, Sami H. A. Almalki, M. Salaheldeen M, Ahmed Alzamil, Md. Shabiul Islam

**Affiliations:** 1 Space Science Center (ANGKASA), Universiti Kebangsaan Malaysia (UKM), 43600 Bangi, Selangor Malaysia; 2 Department of Electrical, Electronic and Systems Engineering, Faculty of Engineering and Built Environment, Universiti Kebangsaan Malaysia, Bangi, Malaysia; 3 Department of Computer and Communication Engineering, Faculty of Computer Science and Engineering, Patuakhali Science and Technology University, Patuakhali, Bangladesh; 4 Department of Electrical Engineering, College of Engineering, Taif University, PO Box 11099, Taif, 21944 Kingdom of Saudi Arabia; 5 Department of Electrical Engineering, Faculty of Energy Engineering, Aswan University, Aswan, 81528 Egypt; 6 Electrical Engineering Department, College of Engineering, University of Ha’il, Ha’il, 81481 Saudi Arabia; 7 Faculty of Engineering, Multimedia University (MMU), 63100 Cyberjaya, Selangor Malaysia

**Keywords:** Metamaterials, Electrical and electronic engineering

## Abstract

This study has investigated the impact of inverse G-like shape resonators that exhibited epsilon negative (ENG) and near-zero refractive index (NZI) properties for multi-band wireless communications applications. The electrical measurement of structure is 0.118λ × 0.118λ × 0.021 λ, which is calculated at 3.94 GHz. FR-4 is used as a substrate layer, and the resonator is designed on it. This structure is manifested in the ENG and NZI characteristics within the frequency range of 3.8–4.17, 7.68–8.54, 10.67–11.36 GHz, and 4.07–4.15 and 8.29–8.37 GHz, respectively. This study also manifests the polarization insensitivity nature of 0°–90°, and the incident angle is investigated up to 60° for both TE and TM modes. The proposed structure achieves triple resonance at 3.94 GHz, 8.08 GHz, and 11.17 GHz, respectively, included in the S, C, and X frequency bands. The CST Microwave Studio 2019 software is conducted to design, develop, perform analysis, investigate electromagnetic properties, and extract effective medium parameters. The Advanced Design Software (ADS) is used to model the equivalent circuit of the unit cell. The simulated, measured, and ADS results verified the scattering parameter performance. The EMR value of the structure is 8.47, indicating the structure's compactness. The compact design with simplicity, ENG, and NZI properties make the proposed structure significant for microwave application, mainly to enhance the antenna bandwidth and gain filter design. ENG and NZI properties the operation frequency stability and efficiency for low earth orbit nanosatellite communications.

## Introduction

Metamaterials contain exceptional electromagnetic properties like negative permittivity or negative permeability.  The SNG metamaterial shows negative properties of permittivity or permeability ^1^. DNG presences some distinctive properties of negative permittivity, negative permeability, and negative refractive index, respectively which can be considered LHM (Left-Handed Metamaterial) or DNG (Double negative Metamaterial) metamaterial^2^. Periodic structure-based artificially composite material exhibits negative index properties and can be customized in shape, size, and substrate^3–5^. Metamaterials application covers the area of UV absorber design^6^. A cross-band metamaterial absorber is designed for GHz and THz frequency bands^7^. A ZnSe-based polarization-independent perfect absorber is designed^8^ for the terahertz frequency regime; this absorber exhibits 98.44% and 98.28% absorbability at two resonance frequencies at the terahertz regime. A metamaterial-based leaky-wave antenna is used in radar communication^9^, bandwidth enhancement for multi-band components^10^, specific absorption ratio (SAR) reduction^11^, etc. Thus, a broad scope of research extends to metamaterial reflector-based microwave sensors^12^. In addition, an electromagnetic wave absorber is convenient for radio communication and intelligent radar communication systems^13^. A closed circular ring resonator-based (CCRR) perfect metamaterial absorber is designed on an FR-4 substrate with a thickness of 1.6 mm and a dimension of 22 × 22 mm. This absorber achieved 100 % absorption at 8.3 GHz, 100% absorption at 9.2 GHz, 98% absorption at 9.88 GHz, 91% absorption at 10.66 GHz, and 98% absorption at 11.16 GHz, and 98% absorption at 11.85 GHz, respectively. Moreover, this structure is shown up to 90° polarization insensitivity response for TE and TM modes^14^. This structure exhibits polarization angle and incident angle insensitivity up to 60^0^. The experimental result supports the simulated this structure has been used as a potential candidate ^15^.

Recently, MTM has been designed to target the frequency bands where S-band, C-band, and X-band frequencies are extended to 2–4 GHz, 4–8 GHz, and 8–12 GHz. S-band frequency can be used in surface ship radar transmission and weather radar, and C-band frequency can be used in radio and satellite communications. X-band frequency can be used in military communication and radar applications^16^. The sol–gel method was used to synthesize the ZnO nanoparticles and investigate the magnetic properties of NixCo1–xFe_2_O_4_. This nanoparticle property covers the X-band frequencies to be used in radar communication, defense automobiles, and aeronautics^17^. A single negative (SNG) MTM unit cell is designed with a dimension of 8 × 8 mm^2^ to escalate the multi-band antenna performance^18^. A spider net-shaped MTM unit cell is designed with a size of 10 × 10 mm^2^, and it has an EMR value of 10.48. This spider net-shaped MTM unit cell is includes  the frequency band of S, C, X, and Ku , respectively^19^. A circularly polarized metamaterial-based dual-band antenna is presented in^20^ for WiMAX and WLAN applications. The structure size is 9 × 9 mm^2^, and the EMR value is 9.95. In^21^, A metamaterial unit cell is designed with a compact size of 5.5 × 5.5 mm^2^ for C-band applications that exhibit DNG characteristics. An MTM-based sensor is designed with a dimension of determines 22.86×10.16 mm2 to regulate the electromagnetic properties of the branded and unbranded fuel in X-band frequencies^22^. Later, a single negative (SNG) metamaterial is considered in^23^ with a measurement of 8 × 8 mm^2^ to apply in the S, C, and X frequency bands. An adapted H-shaped MTM is presented in^24^, but this structure shows considerable EMR value with a dimension of 9 × 9 mm^2^. The symmetric resonator-based MTM unit cell is designed in^25^; this structure shows the ENG and NZI properties. A gap coupled hexagonal-shaped MTM unit cell is introduced in^26^ to apply in the S-band and X-bands microwave frequencies . This unit cell shows the effective medium value of 8.40 with a measurement of 10 × 10 mm^2^. A split-ring resonator (SRR) significantly affects the resonance frequency of MTM-based geometrical unit cells, and this symmetrical structure helps achieve the polarization independence characteristics^27^. In addition, A single layer MTM unit cell is designed to operate in X and Ku microwave frequency bands, as well as this structure, is investigated the polarization-dependent response up to 90°^28^.

This study has designed an inverse G-like shape MTM unit cell to achieve the targeted resonance frequency of 3.94 GHz, 8.08 GHz, and 11.17 GHz.The selected resonance frequency is included in the S, C, and X-band for satellite and radar communication applications. The structure manifests ENG characteristics in the frequency range of 3.8–4.17, 7.68–8.54, 10.67–11.36 GHz, and NZI is exhibited in 4.07–4.15 and 8.29–8.37 GHz. The CST Microwave Studio 2019 software designs the structure with a compact dimension of 9 × 9 × 1.6 mm3, and ADS 2020 software is used to develop the equivalent circuit of the structure. This study manifests the polarization insensitivity nature (0°–90°) and the response of incident angle stability (0°–60°) for TE and TM propagation modes. The structure has potential application in microwave wireless communication since it has a compact size with design simplicity. The proposed unit cell can be used to increase the antenna bandwidth and filter design since it exhibits ENG properties and enhances an antenna's gain performance since it exhibits NZI properties. In addition, the operating frequency stability enables the structure to use in low earth orbit nanosatellite communications^29^. The unit cell design structure and simulation setup are characterized in section two then the design evaluation is analyzed and developed toward the proposed MTM unit cell. Section three investigates the Electric field (E), magnetic field (H) intensity, then the surface current behavior (density and circulation) at different frequencies. Finally, section four is discussed the equivalent circuit designing. The extraction method of effective medium parameters, polarization and incident angle stability, the experimental verification , discuss the simulated and measured result and calculate EMR in section five.

## Design procedure and simulation setup of the structure

**Figure 1:**
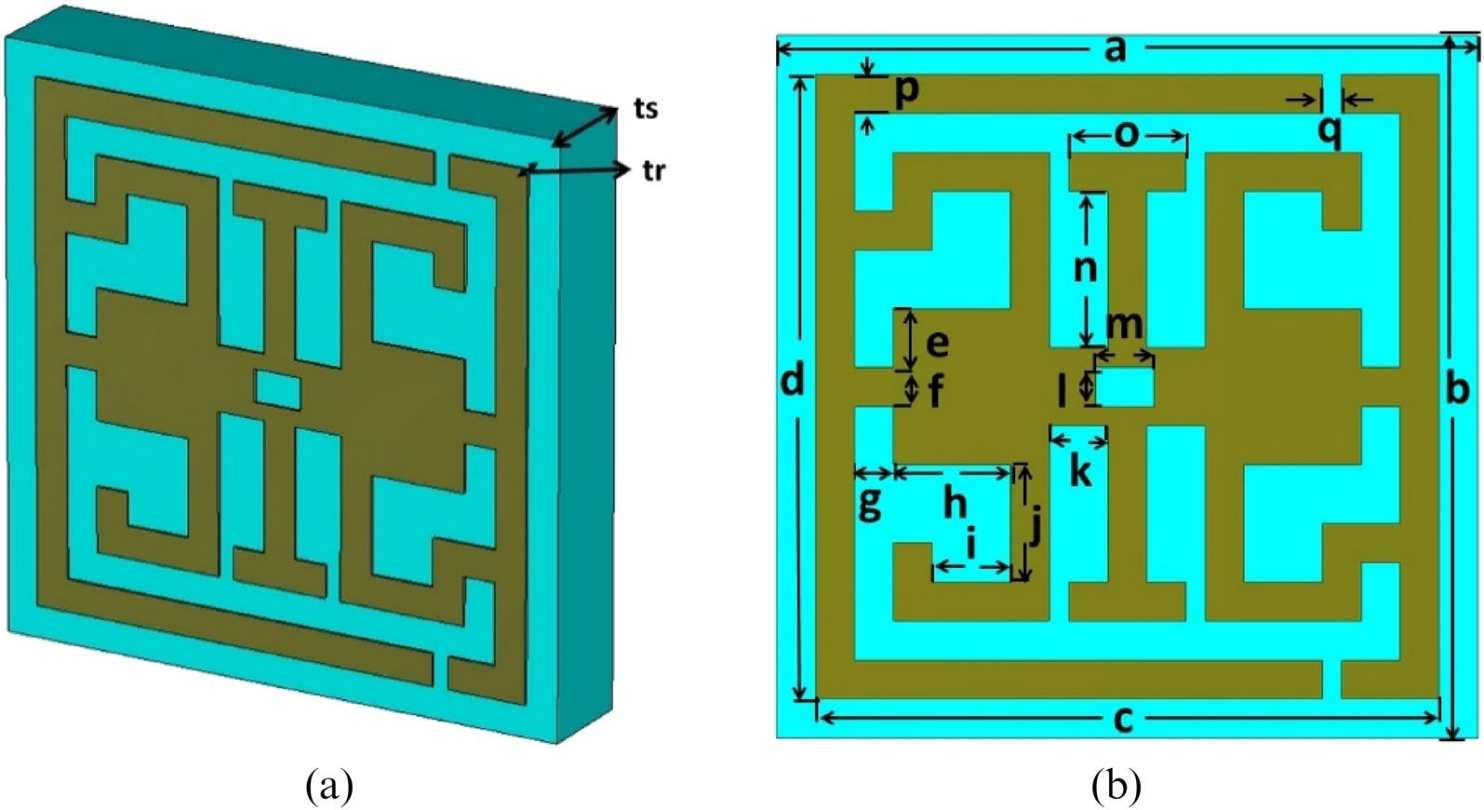
(**a**) Anticipated structure  (**b**) Structural layout of the anticipated structure (CST STUDIO SUITE 2019, https://www.3ds.com/products-services/simulia/products/cst-studio-suite)^30^.

**Figure 2:**
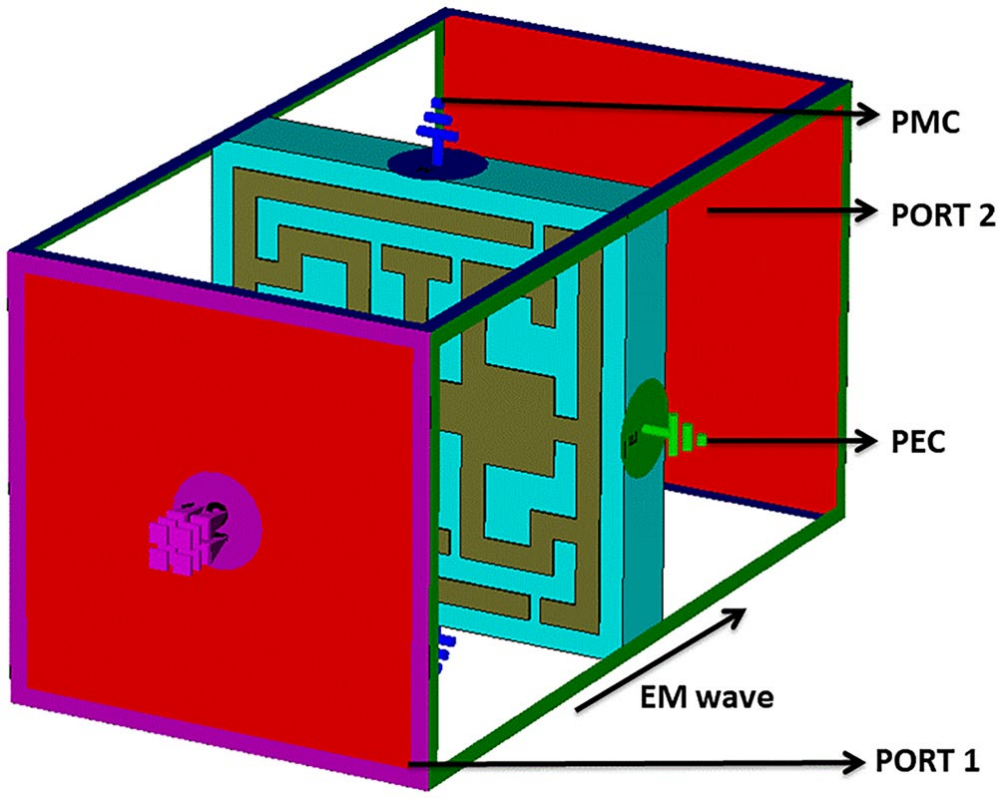
Anticipated structure with simulation setup (CST STUDIO SUITE 2019, https://www.3ds.com/products-services/simulia/products/cst-studio-suite)^30^.

The anticipated structure is designed with a compact dimension of 0.118λ × 0.118λ × 0.021 λ; λ is calculated at a 3.94 GHz lower resonance frequency. The anticipated structure is designed on a 1.60 mm FR-substrate; the loss tangent and dielectric constant values of FR-4 are 0.02 and 4.4. The perfect electric conductor (PEC) is used to design the resonator of the structure. The anisotropic structure is presented in Fig. 1a, and the layout of the designed resonator is depicted in Fig. 1). The structure contains an I-like shape at the center of the resonator, two G-like shapes inversely placed at the sides of the I-like shape, and SSRR, all of the resonating elements adjacent to the metal line.  The dimension of the I-like shape is significant  in shifting the structure's resonance frequency.The trial and error method is used to choose different split gaps of the resonator so that the unit cell can provide maximum resonance. Table 1 is presented all of the necessary parameters with their values. The CST studio suite 2019 is numerically performed over 3–12 GHz. The transmission and reflection coefficient (S_11_, S_21_) is observed to ensure effective responses included in the S, C, and X frequency bands. Figure 2 is depicted the structure simulation setup . The X-axis and Y-axis are instigated as PEC and PMC, where all the planes are perpendicular to each other. The electromagnetic field is applied through Z-axis.

**Table 1:** Design parameters of anticipated structure with their value .

Parameter	Dimension (mm)	Parameter	Dimension (mm)
*a*	9	*k*	0.75
*b*	9	*l*	0.60
*c*	8	*m*	0.75
*d*	8	*n*	2
*e*	0.75	*o*	1.5
*f*	0.50	*p*	0.50
*g*	0.50	*r*	0.25
*h*	1.50	*tr*	0.4
*i*	1	*ts*	1.57
*j*	1.50

### Development of the anisotropic structure 

**Figure 3:**
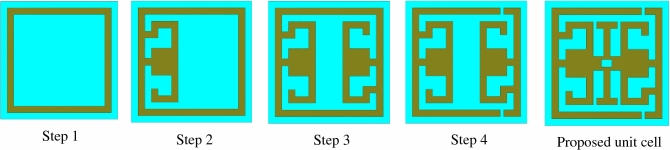
Design evaluation of the structure (CST STUDIO SUITE 2019, https://www.3ds.com/products-services/simulia/products/cst-studio-suite)^30^.

**Figure 4:**
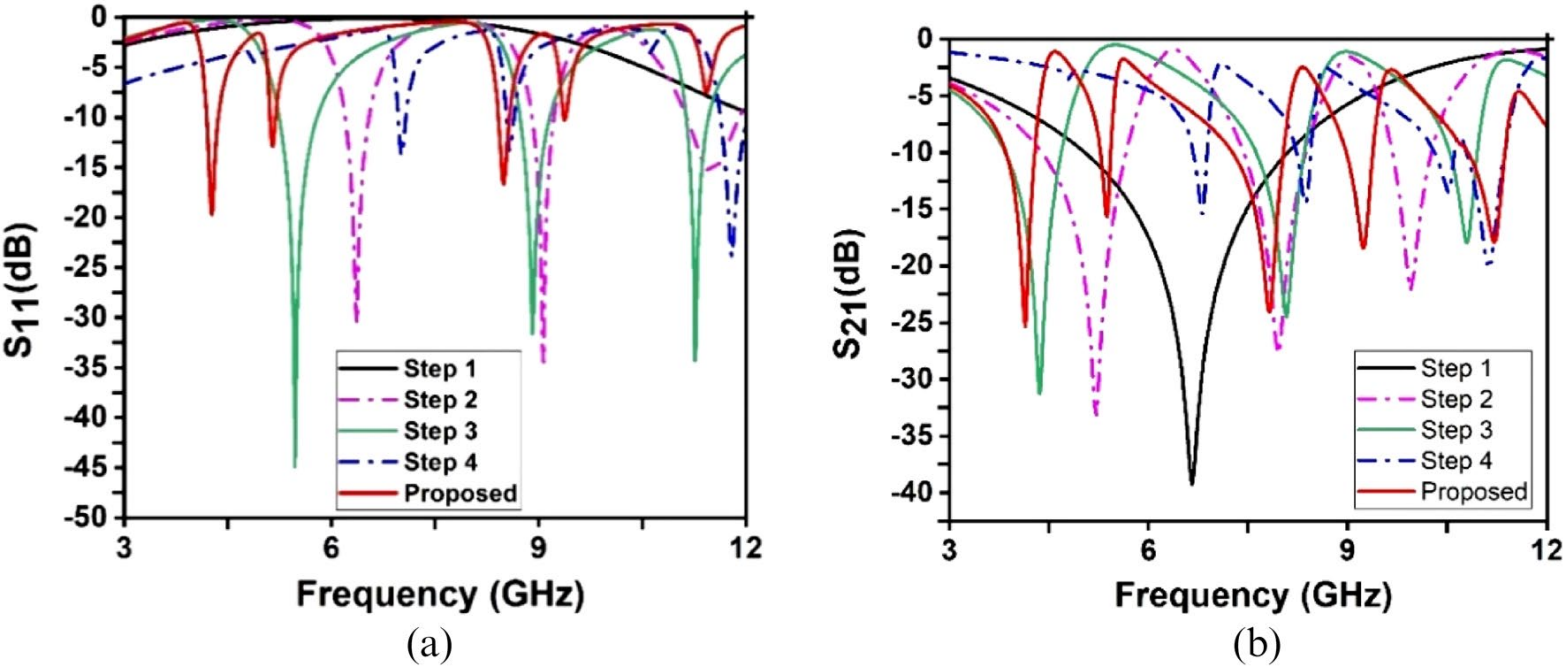
Scattering parameters of evolution steps toward the designed metamaterial unit cell: (**a**) reflection coefficient (S_11_) (**b**) transmission coefficient (S_21_).

Several types of design are investigated in this study to get the electromagnetic properties of anticipated structure the. Figure 3 is demonstrated the different configurations of the structure, where Fig. 4a and b are shown the response of S_11_ and S_21_, respectively. In step1, a square ring resonator (SRR) is designed, for instance, to provide a single resonance of S_21_ at 6.66 GHz, as shown in Fig. 4b. In step 2, an inverse G-like shape is loaded at the left position of the SRR; this configuration subsidizes two resonances of S_11_ at 6.30 GHz and 9.10 GHz, as shown in Fig. 4a, where the resonance of S_21_ is located at the frequency of 6.36 GHz and 9.05 GHz, respectively is shown in Fig. 4b. Then metallic strip connects the inverse G-like shape and SRR. In step 3, another G-like shape is loaded at the right portion of the SRR, which causes shift resonances of S_11_ and S_21_toward the lower frequency at 5.40 GHz and 5.47 GHz, presented in Fig. 4a and b. After that, in step 4, two split gaps are introduced at the top and bottom of the right corner of the SRR; this modification causes a resonance shift towards the higher frequency at 11.97 GHz that covers the X frequency band shown in Fig. 4b. Later, an I-like segment is designed at the resonator's center, interconnected between two G-like shapes. The length of the I-like shape plays a vital role in increasing the electrical length and shifting the resonances frequency. These I-like segment causes earlier shift resonance of S_11_ at 4.25 GHz and resonance of S_21_ at 4.27 GHz, respectively, are shown in Fig. 4a and b. All shapes are interconnected by adding two square-shaped metallic strips on two sides of the I-like shape. These two square-shaped metallic strips allow the current to flow from one G-like shape to another under the effect of H-field. Finally, a parametric sweep is performed to achieve lower resonance at the 3.09 GHz, including the S-band frequency range shown in Fig. 4b. Table 2 summarizes the response of S_21_.

**Table 2:** Transmission Coefficient (S_21_) of successive steps for the proposed unit cell.

Substructure	Resonance frequency (GHz)	Resonance peak (dB)	Covering bands
Step 1	6.66	− 39.37	C
Step 2	6.36, 9.05	− 30.54, − 33.39	C
Step 3	5.47, 8.91, 11.26	− 44.96, − 31.64, − 34.28	C, X
Step 4	7.04, 8.57, 11.97	− 12.77, − 13.27, − 23.89	C, X
Proposed unit cell	3.94, 8.08, 11.17	− 25.10, − 18.44, − 17.88	S, C, X

## E-field, H-field, and surface current analysis

**Figure 5:**
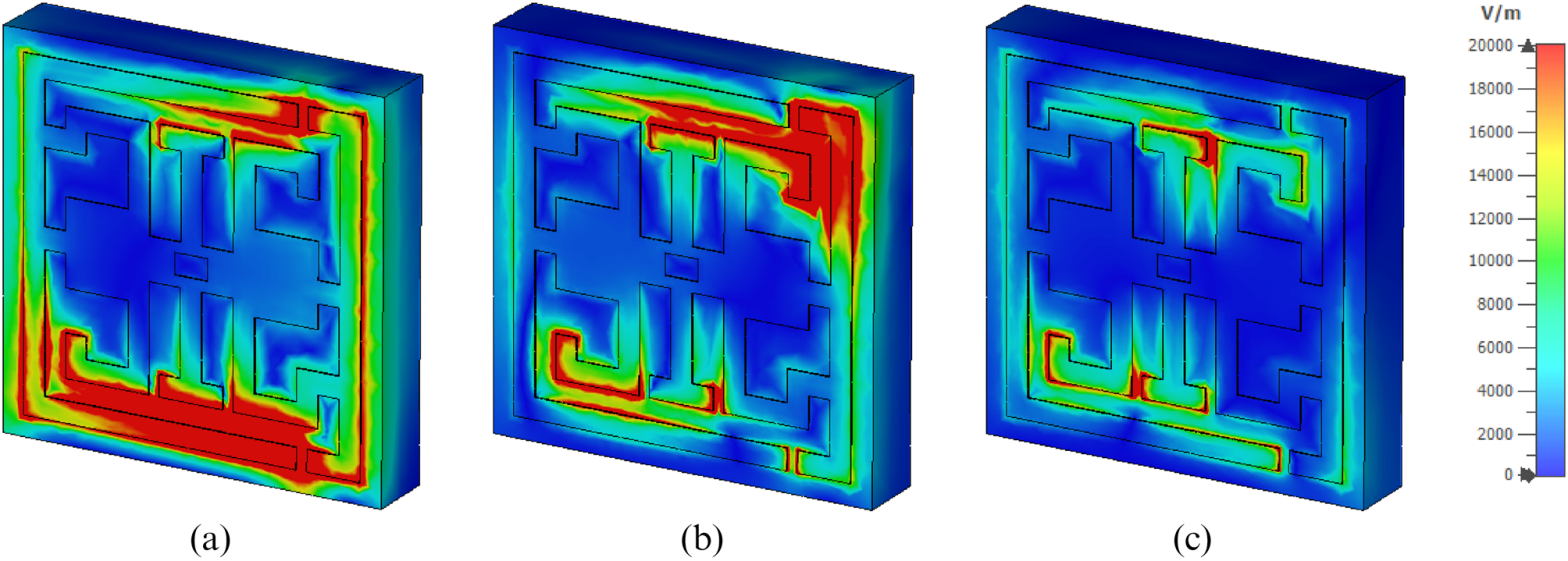
Electric field (**a**) 3.94 GHz, (**b**) 8.08 GHz, (**c**) 11.175 GHz (CST STUDIO SUITE 2019, https://www.3ds.com/products-services/simulia/products/cst-studio-suite)^30^.

The analysis of E-field, H-field, and surface current distribution at  different resonance frequencies help to understand the structure. The electromagnetic waves penetrate the unit cell during the simulation process and respond to S_11_ and S_21_. At 3.94 GHz frequency, a high-intensity E-field is observed at the bottom and top resonator's corners, as displayed in Fig. 5a. The higher density, lower high, medium, lower medium, then less intensity of the E-field, H-field, and surface current are indicated by red, orange, yellow, green, and blue colors. The design saw medium E-field intensity at the lower portion of the inverse G-like shape, inverse G-like shape, and I-like shape. Moreover, the same phenomenon has happened in the top and bottom parts of the I-like segment shown in Fig. 5b. On the other hand, at 11.175 GHz, the strength of the electric field (E) over the entire resonator is decreased, and a lower electric field (E) intensity is observed over the SSRR.

**Figure 6:**
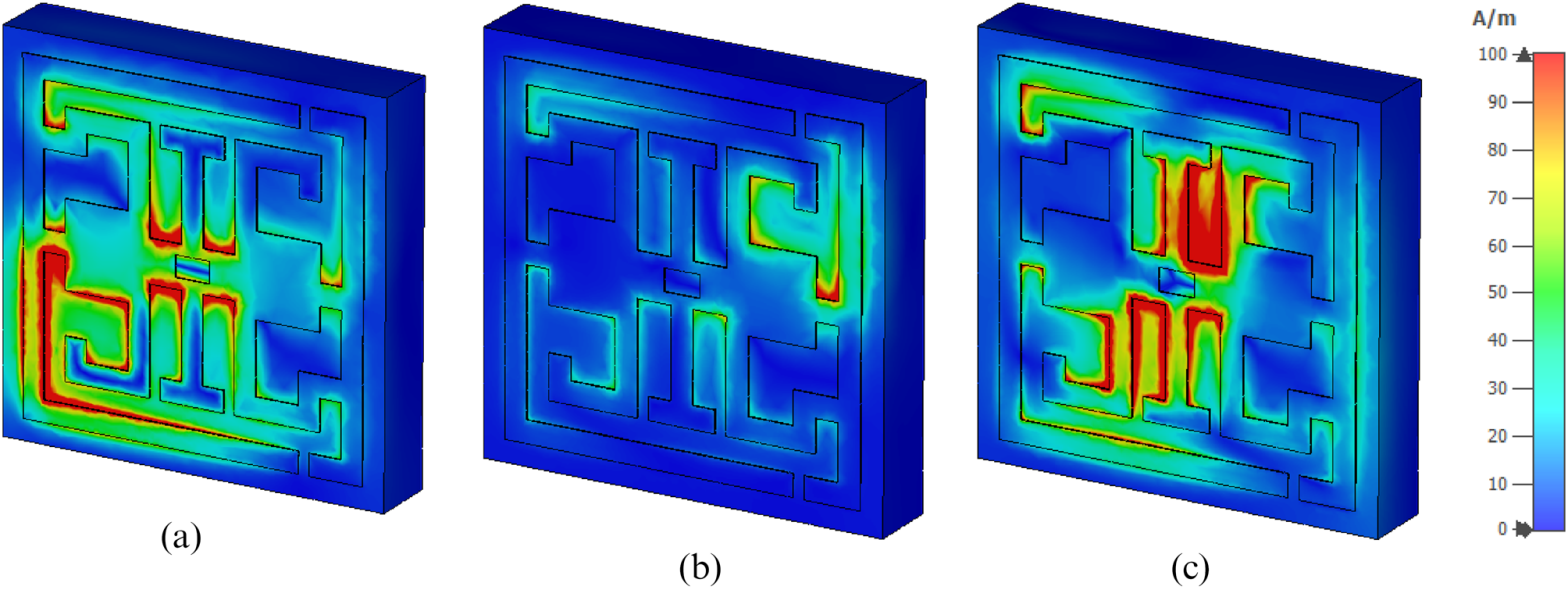
Magnetic field (**a**) 3.94 GHz, (**b**) 8.08 GHz, (**c**) 11.175 GHz (CST STUDIO SUITE 2019, https://www.3ds.com/products-services/simulia/products/cst-studio-suite)^30^.

**Figure 7:**
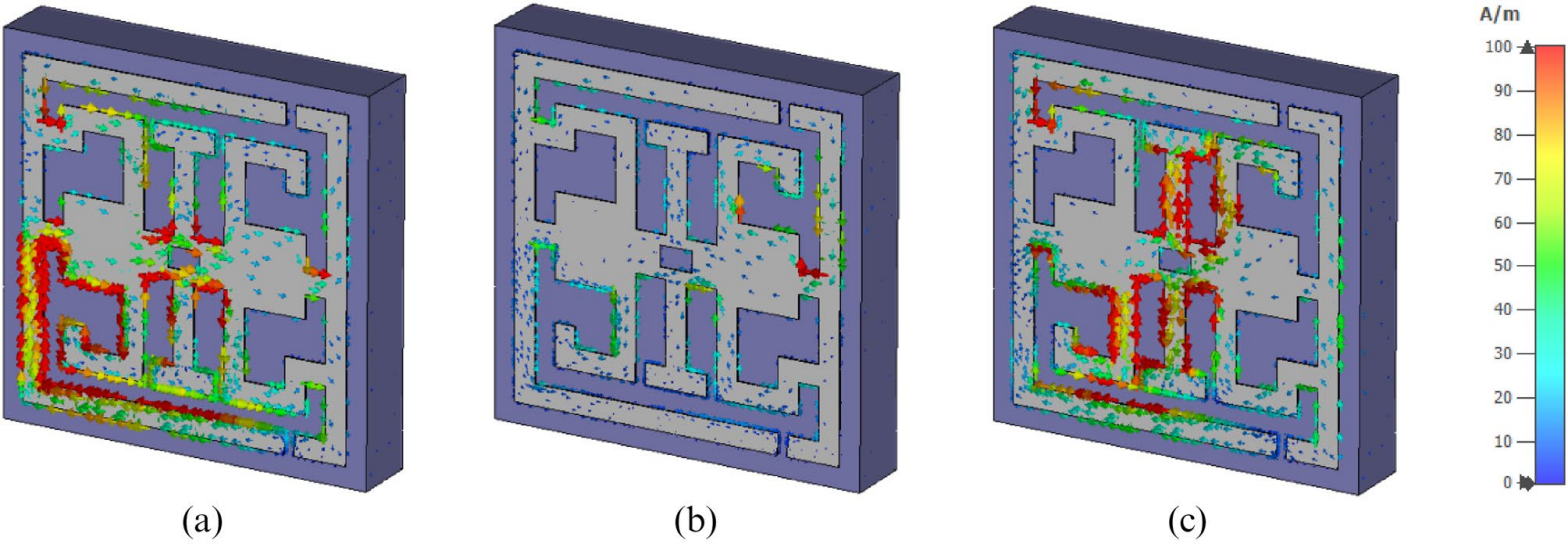
Surface current (**a**) 3.94 GHz, (**b**) 8.08 GHz, (**c**) 11.175 GHz (CST STUDIO SUITE 2019, https://www.3ds.com/products-services/simulia/products/cst-studio-suite)^30^.

Nevertheless, a comparatively high intensity electric field (E) is perceived  at the top and bottom of the I-like shape and then at the bottom of inverse G-like shape, as displayed in Fig. 5c. At 3.94 GHz, an energetic magnetic field (H) intensity is observed at the middle and lower left portions of the inverse G-like shape. A relatively lower magnetic field (H) intensity is noticed at the top and bottom right parts of the resonator, as depicted in Fig. 6a. Dramatically magnetic field intensity is decreased in the resonator at 8.08 GHz except for the top right portion, as presented in Fig. 6b. However, High magnetic field intensity is observed around the I-like shape at 11.175 GHz, as illustrated in Fig. 6c. Later, the circulation and distribution of surface current are analyzed.A strong surface current is observed at 3.94 GHz in the lower-left portion of the inverse G-like shape. The medium surface current intensity is observed in the middle of the I-like shape. Low, medium intensity is observed at the lower portion of the SSRR presented in Fig. 7a. Consequently, a well-distributed surface current is observed at the entire resonator at 8.08 GHz. Still, a strong current is observed in the middle right part of the resonator, presented, in Fig. 7b. A strong surface current distribution was at 11.75 GHz observed around the I-like shape and bottom portion of the inverse G-like shape, as presented, in Fig. 7c.

## Equivalent circuit modeling

The metamaterial structure contains metal strips with split gaps that exhibit inductive and capacitive effects. The metal strips induce the inductance, whereas split gaps contribute to the capacitance of the structure. Thus, the whole structure resembles an LC circuit that exhibits resonance, with resonance frequency (*f*) having the following relation^31^:

1 $$f = \frac{1}{{2\pi \sqrt {LC} }}$$

Here, L & C represent the inductance and capacitance. The split gap and metal strips have a capacitive and inductive effect. The split gap can be determined by using the relation as expressed in Eq. (2):

2 $$C = \varepsilon_{0} \varepsilon_{r} \frac{A}{d}\left( F \right)$$

here, $\varepsilon_{0}$ and $\varepsilon_{r}$ denote the free space permittivity and relative permittivity, whereas A and d indicate the metal strip area and split distance. The inductance of the resonator ring can be computed by applying the transmission line principle^32^:

3 $$L\left( {nH} \right) = 2 \times 10^{ - 4} l\left[ {\ln \left( {\frac{l}{w + t}} \right) + 1.193 + 0.02235\left( {\frac{w + t}{l}} \right)} \right]K_{g}$$

**Figure 8:**
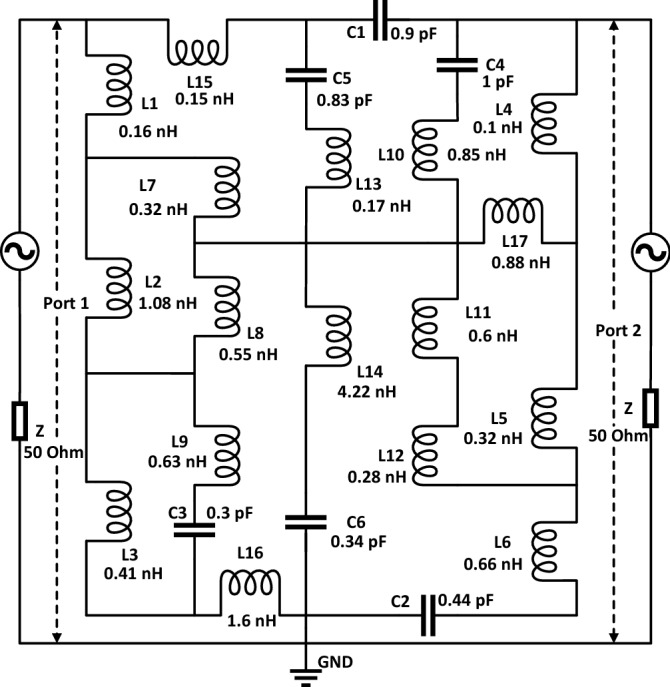
Equivalent circuit of the unit cell.

**Figure 9:**
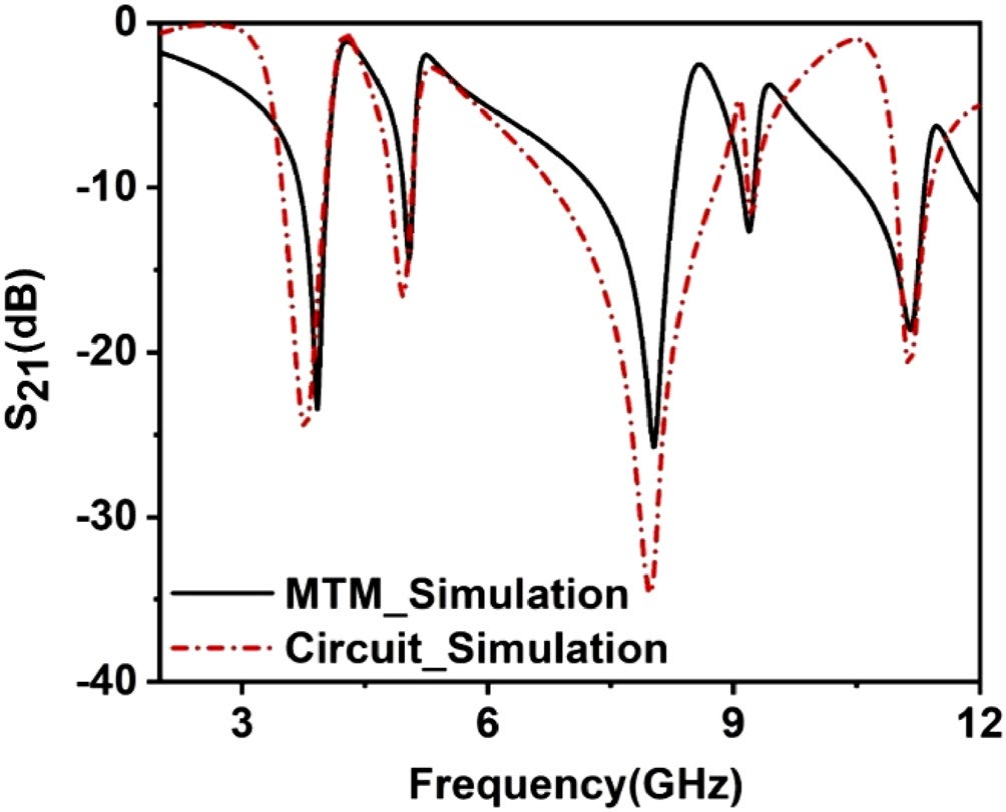
Transmission coefficient obtained from the equivalent circuit simulated in ADS and CST simulation.

Here, the correction factor, $K_{g} = 0.57 - 0.145 \ln \frac{{w^{\prime } }}{{h^{\prime } }}$ , in which $w^{\prime }$ and $h^{\prime }$ are represent the substrate width and thickness, respectively. Moreover, $t, l,$ and $w$ denote the thickness, length, and width of microstrip lines, respectively. The projected MTM structure conceives a square outer ring that is split twice. An equivalent circuit is considered in Fig. 8, in which this outer ring is presented by a group of inductances and capacitances, namely *L1*, *L2*, *L3*, *L4*, *L5*, *L6*, *L15*, *and L16*. This inductance resembles the effects of copper for different segments of the outer rings, whereas capacitors *C1* and *C2* are due to the split gaps in this ring. This ring is an interconnector with two modified E-shaped metal strips that are presented with the equivalent inductances *L7*, *L8*, *L9*, *L10*, *L11*, *L12* in distribution form like the line inductances in a transmission line. The middle of the I-like-shaped structure equates with the inductance L13, L14, and as the I-like shape is connected with two E-shaped structures, a short circuit is employed in the middle of the equivalent circuit. Moreover, inductance *L17* characterizes the corresponding inductance between E outer ring and internal structure. The co-planar capacitances that exist between the outer ring and internal structures are symbolized by the capacitances *C3*, *C4*, *C5,* and *C6*. The equivalent circuit is justified through the simulation in Advanced Design System (ADS) and comparing the result obtained from this with the output obtained in CST microwave studio. In ADS, two ports are connected at two ends of the circuit, including 50 Ω terminating impedances, as shown in Fig. 1. Initially, component values are considered 1 nH for each inductor and 1 pF for each capacitor. Then, the component values are adjusted using the tuning module of ADS to attain the similar S_21_ response of MTM simulation in CST, and thus, the component values are finalized. Figure 9 shows a comparison plot of S_21_ that describes the nature of the transmission coefficient acquired from circuit simulation and MTM simulation. A close similarity is noticed between the two responses; thus, the equivalent circuit in Fig. 1 with component values replicates the MTM unit cell present in this manuscript.

## Results and discussion

### Extraction method of effective medium parameters

**Figure 10:**
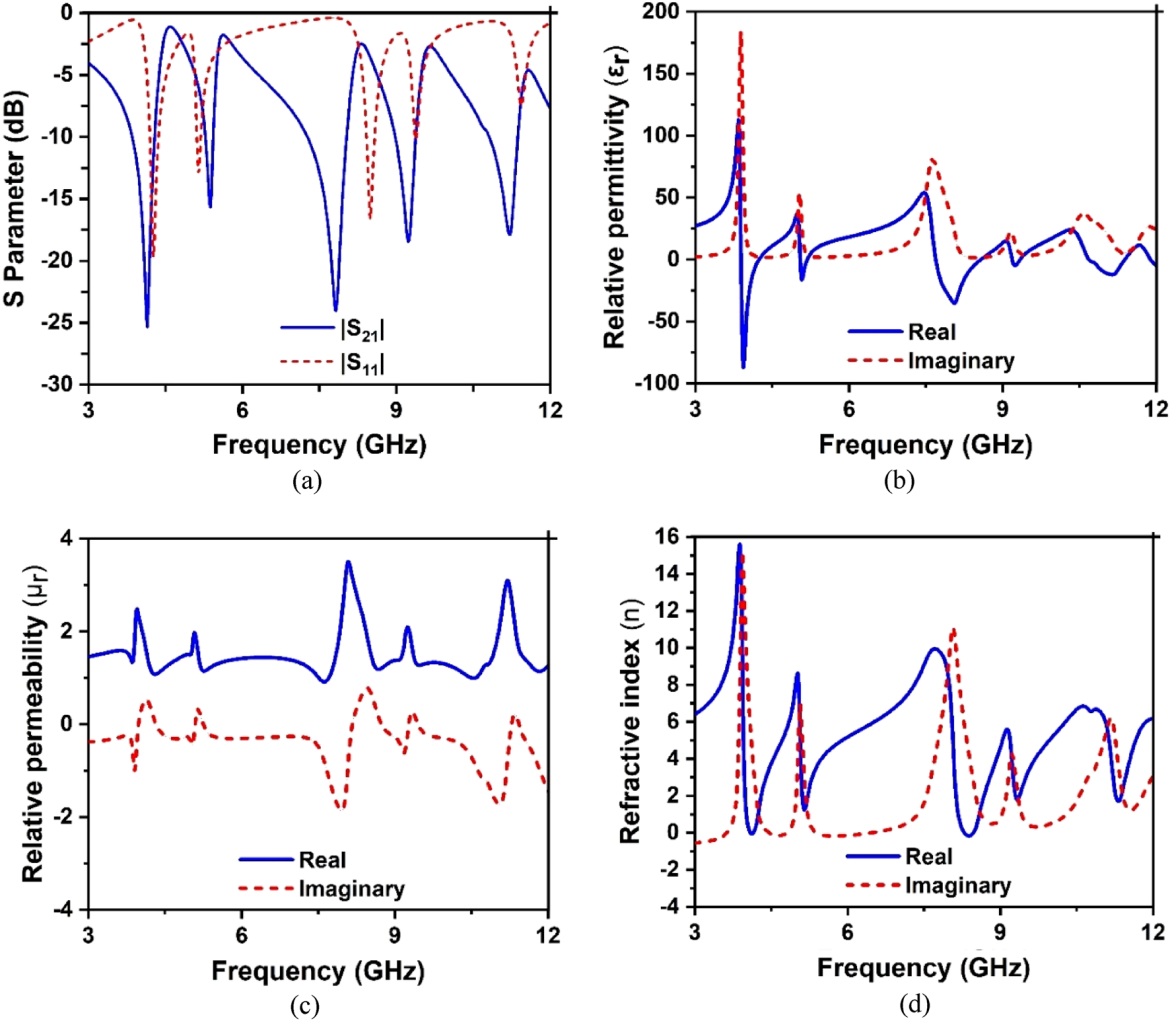
Scattering parameters for evolution steps towards the proposed unit cell: (**a**) S-parameter, (**b**) relative permittivity, (**c**) relative permeability, and (**d**) refractive index.

The graphical depiction of S-parameters adapted with the CST post-processing section is demonstrated in Fig. 10a–d. The robust retrieval method is used in CST 2019 software to extract and explain the S-parameter and effective parameters. The unit cell is shown three resonance of S_21_ at 3.94 GHz, 8.08 GHz, and 11.17 GHz with an amplitude of − 25.10 dB, − 18.44 dB, and − 17.88 dB, respectively. Conversely, the resonance of S_11_ at 3.97 GHz and 8.49 GHz with an amplitude of − 20.24 dB and 17.13 dB, respectively. Resonance of the reflection coefficient (S_11_). Fig. 10a has depicted the response of S11 and S21 to the structure. Each lower resonance value of S21 is consistently lower than the corresponding resonance of S11. Regarding data of S-parameters is presented in Table 3. The negative relative permittivity characteristic is exhibited at 3.8–4.17 GHz, including the S-band frequency shown in Fig. 10b.

**Table 3:** Response of S21, permittivity, and refractive index of the structure.

Parameter	Frequency range (GHz)	Extracted property
Transmission coefficient (S_21_)	3.82–4.25, 7.26–8.03, 10.13–11.36	|S_21_|< − 10 dB
Permittivity, (ϵ)	3.8–4.17, 7.68–8.54, 10.68–11.38	ϵ < 0
Nera Zero Refractive index, (n)	4.07–4.15 8.29–8.37	*n* ≈ 0

After that, another negative relative permittivity characteristic is exhibited between the 7.68–8.54 GHz frequency ranges. Moreover, in the rest of these frequency ranges, the unit cell exhibited negative permittivity values between the 5.04–5.17 GHz frequency ranges with slightly lower amplitude value including C-band frequency.  Furthermore, another negative permittivity is exhibited in the 10.67–11.36 GHz frequency range, including the X-band frequencies. It is observed from Fig. 10b that when the resonance of S21 occurs at that point, negative permittivity is achieved. It is observed from  Fig. 10b that when the resonance of S21 occurs at that point, negative permittivity is achieved. Moreover, noticed that where the permittivity fluctuates positive to negative then  permeability is positive with a continuous change within the frequency ranges, as shown in Fig. 10c. Figure 10c is presented the graphical representation of relative permeability. The near-zero refractive index is exhibited at 4.07–4.15 and 8.29–8.37 GHz, respectively, shown in Fig. 10d. Since the structure exhibits the negative permittivity property, the structure  can be defined as ENG metamaterials. The SNG metamaterial can be used in several microwave applications, for example, to increase the antenna gain and bandwidth and filter designing^29^.

The response of S_11_ and S_21_ impacts the impedance of structure can be the relation between scattering parameters and effective input impedance Eq. ^33^.

4 $$z_{ in} = \pm \sqrt {\frac{{\left( {1 + S_{11} } \right)^{2} - S_{21}^{2} }}{{\left( {1 - S_{11} } \right)^{2} - S_{21}^{2} }}}$$

The characteristics of impedance,

5 $$z_{0} = \sqrt {\frac{{\mu_{0} }}{{\varepsilon_{0} }}} = 377\;{\Omega }$$

6 $$Z = \frac{{z_{in} }}{{z_{0} }}$$

**Figure 11:**
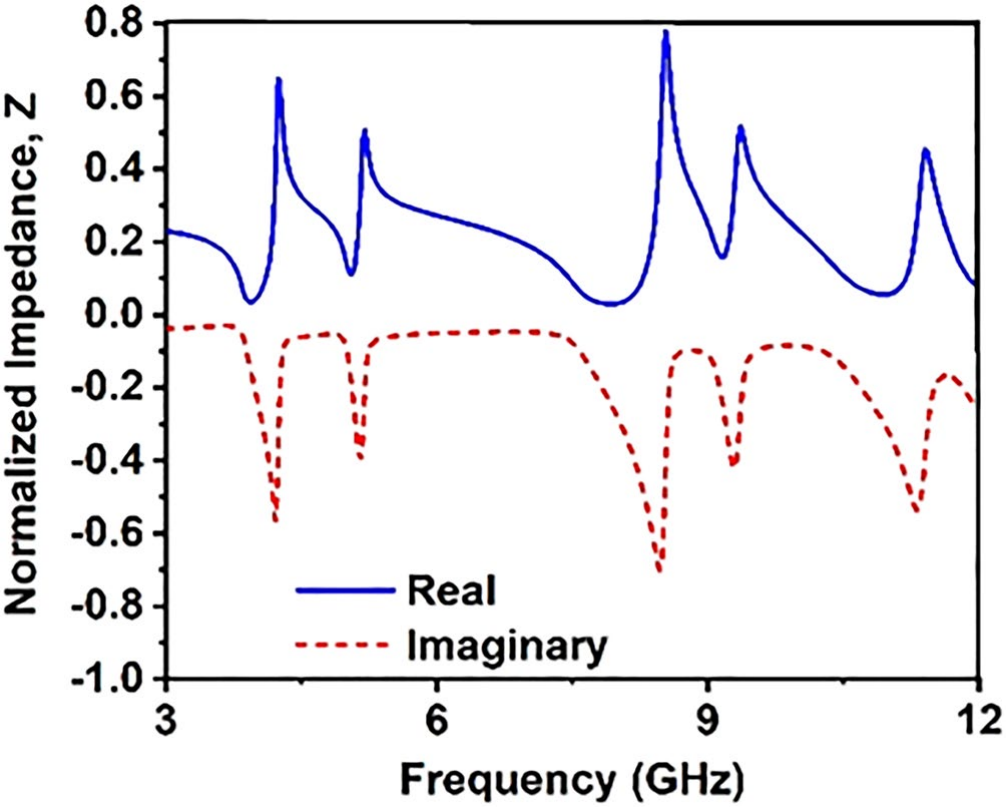
Impedance curve of the structure .

The normalized impedance diagram of the structure is depicted in Fig. 11. As depicted in Fig. 11, the real part of the normalized impedance is positive, whereas the imaginary part is negative. The real part of the impedance approaches near zero in the vicinity of the frequency 3.93 GHz, 8 GHz, 10.94 GHz with a magnitude of 0.035, 0.03, 0.05, respectively. In contrast, two other dips of impedance spectra are observed at 5.04 GHz and 9.15 GHz with a magnitude of 0.11 and 0.15. The positive real part of impedance indicates that forward wave propagation occurs through the proposed MTM when exposed to electromagnetic waves^34^.

### Study the effect of polarization and incident angle change

**Figure 12:**
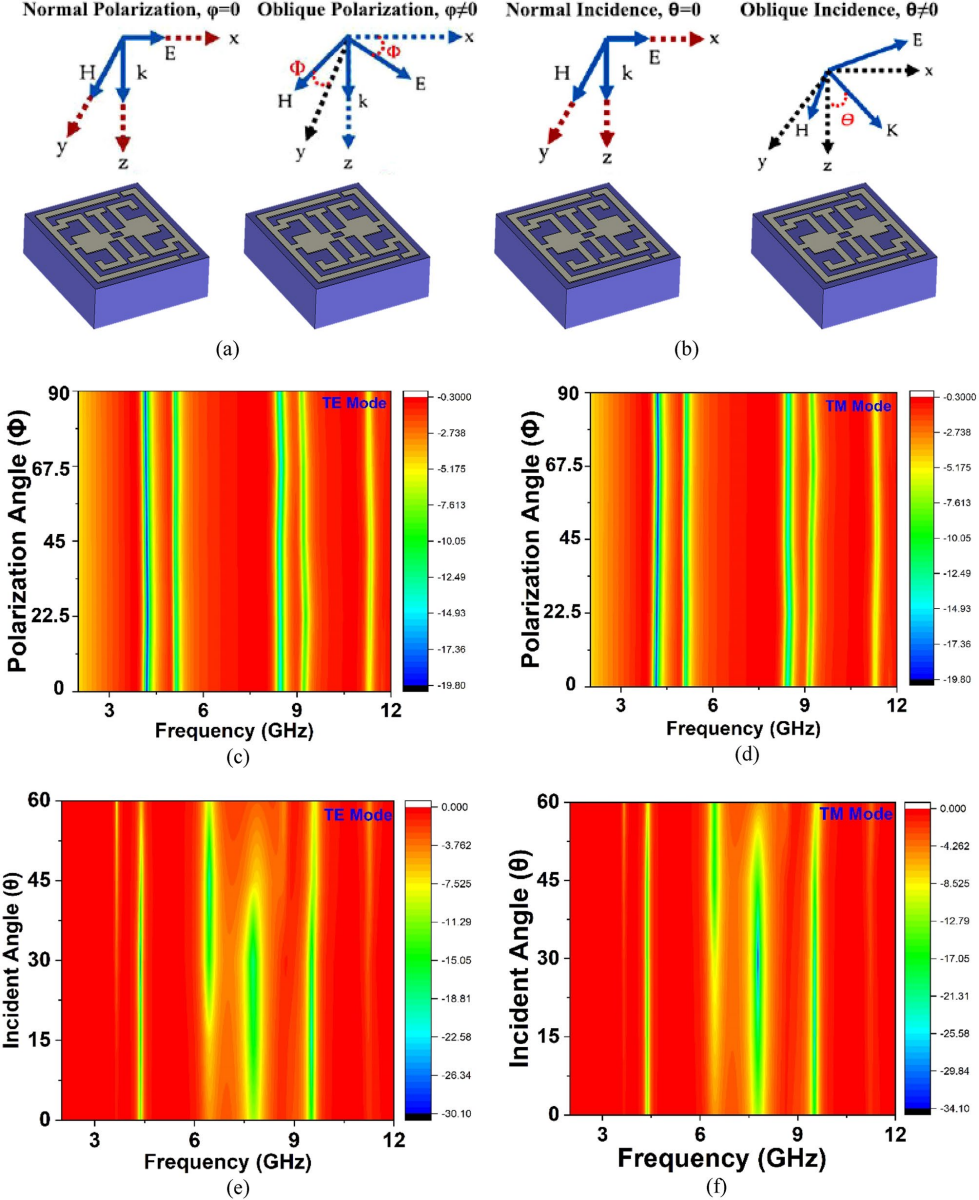
Presentation of (**a**) normal and oblique polarization angle, (**b**) normal and oblique incident angle, (**c**) polarization angle at TE mode, (**d**) polarization angle at TM mode, (**e**) incident angle at TE mode (**f**) Incident angle at TM mode (CST STUDIO SUITE 2019, https://www.3ds.com/products-services/simulia/products/cst-studio-suite)^30^.

This study investigated the changing effect of polarization and incident angle of the structure. The normal polarization angle and oblique polarization are presented in Fig. 12a and b is depicted the normal incident angle and oblique incident angle. The proposed structure is demonstrated  the polarization insensitivity nature (0°–90°) at TE and TM mode, which is presented in Fig. 12c–d. Incident angle (*θ*) is investigated at 0°–60° for TE and TM modes, as displayed in Fig. 12e–f. The incident angle insensitivity is proportion to the path length, so more incident angle insensitivity can be achieved when path length is increased. Moreover, an inclusive electric field is perpendicular to the incident angle for better stability. However, the proposed structure has an excellent coupling effect with a wide polarization angle that increases the application necessities in the communication systems.

### Experimental result analysis of MTM unit cell

**Figure 13:**
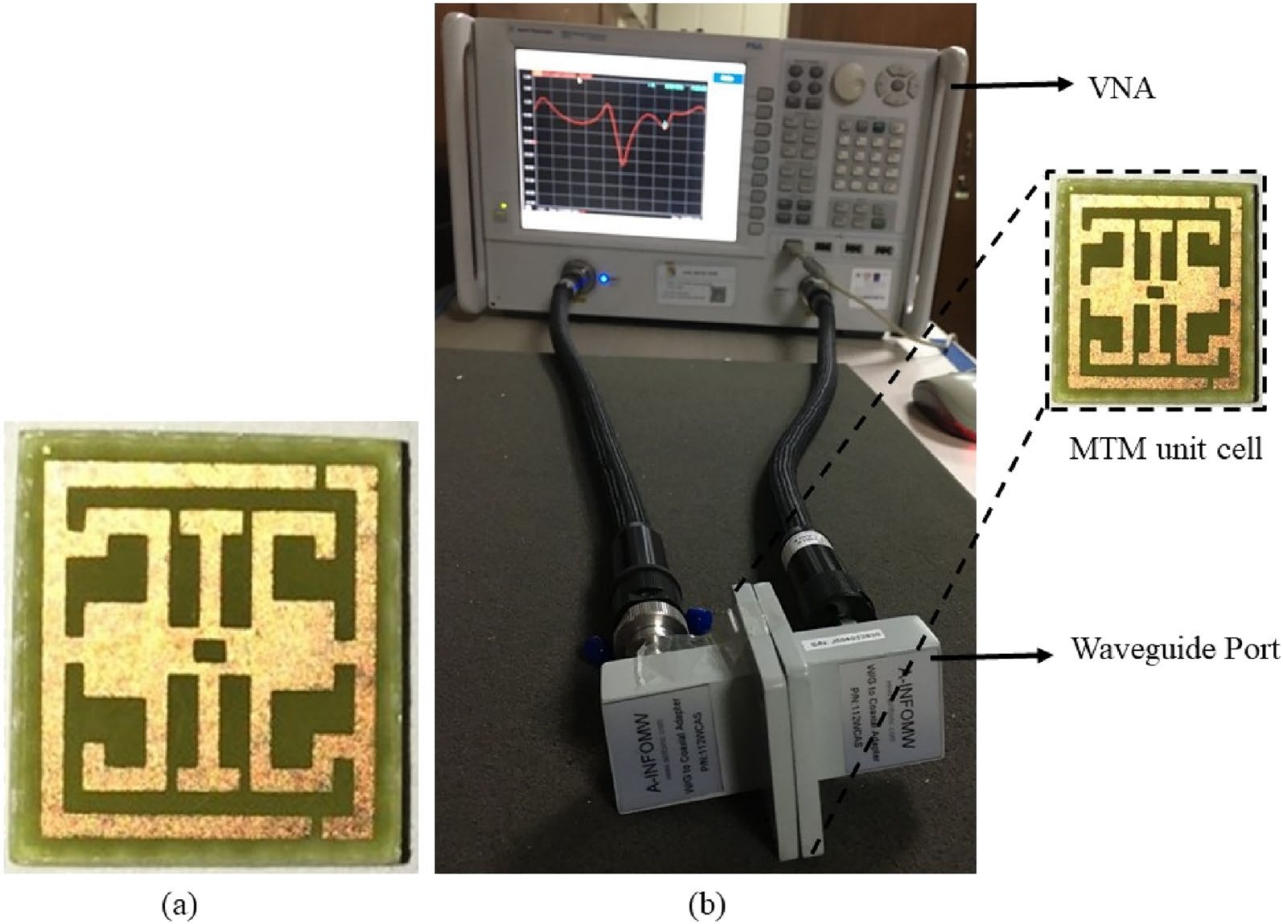
(**a**) Fabricated prototype of structure, (**b**) VNA setup for measurement.

**Figure 14:**
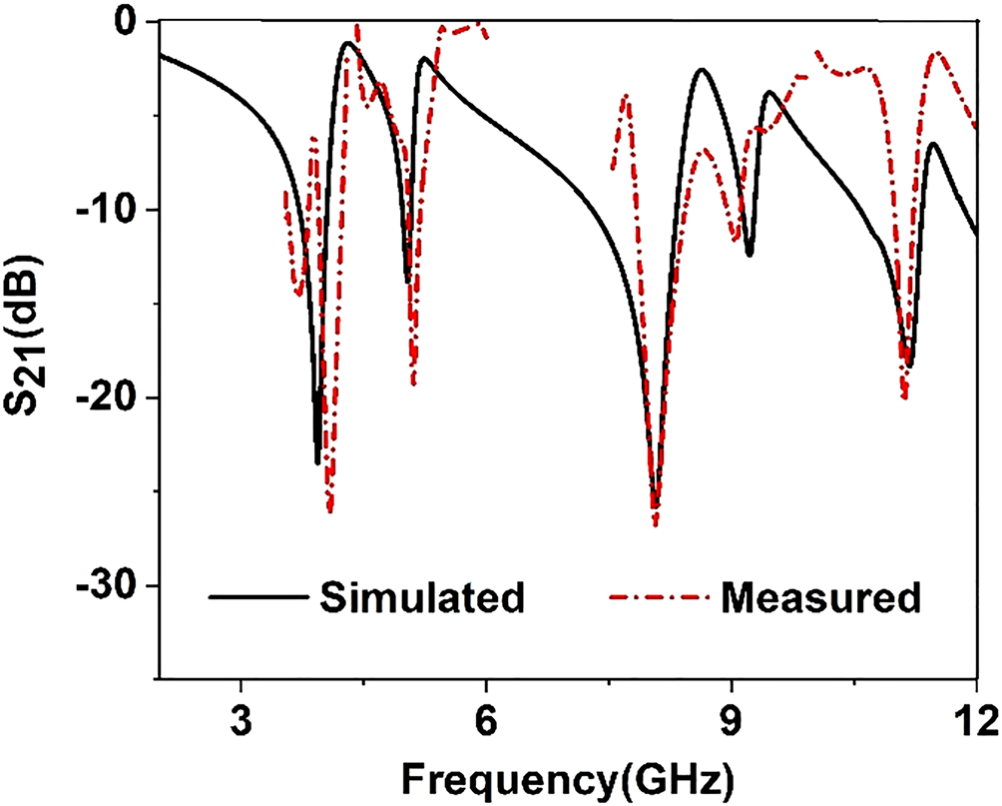
Simulated and Measured result of S_21_.

The structure  is fabricated on a 9 × 9 mm^2^ FR4 substrate to validate experimentally. The transmission coefficient (S_21_) is generated to verify the numerical result from the measurement process. The performance of the fabricated prototype is experimentally verified by using Agilent PNA Network Analyzer N5227A with three different waveguide ports, as shown in Fig. 13a. The prototype is placed between two waveguides to coaxial adapter P/N: 187 WCAS when measuring the transmission coefficient (S_21_) from 3.95–5.85 GHz. Similarly, WG to coaxial adapter P/N: 137 WCAS and WG to coaxial adapter P/N: 75WCAS are used to measure 7–10 GHz and 10–15 GHz, respectively, shown in Fig. 13b. The proposed structure achieves triple resonance during the simulation process at 3.94 GHz, 8.08 GHz, and 11.17 GHz, respectively, as shown in Fig. 14. The measured result also covers these triple resonances at the frequency of 3.96 GHz, 8.18 GHz and 11.20 GHz, respectively, shown in Fig. 13. The first resonance is a slight right shift from 3.94 to 3.96 GHz with an amplitude of − 23.56 dB and − 26 dB. Harmonic resonance is observed in simulated and measured results at 5.10 GHz with − 13.7 dB and − 19.34 dB amplitude, respectively, presented in Fig. 13. Compared to the simulation results, slight differences are observed because of these constraints; the experimental result shows a minor shifting in resonant frequency with harmonics. There are various reasons behind this mitch match, such as fabrication error, drift error, EMI noise, and signal leakage and reflection. However, simulated and measured results included the S, C, and X band frequency range.

### EMR and comparative study of the proposed unit cell

Table 4 compares the proposed structure with existing work  regarding the resonance frequency of transmission coefficient (S_21_), operating band, EMR value, and the physical and electrical dimensions. EMR has significance in controlling the miniaturization of the structure . The EMR is calculated  by using the relation, ${\text{EMR}} = \frac{{\uplambda }}{L}$ Where λ represents the wavelength , and $L$ denotes the structural length of the square-shaped unit cell. The SNG or DNG characteristics can be possible if the EMR is more than 4. The EMR value of the proposed MTM unit cell is 8.47 at 3.94 GHz resonance frequency.The electrical measurement of structure is 0.118λ × 0.118λ × 0.021 λ. From Table 4, it is observed that the dimension of Ref.^21^ and Ref.^24^ is less than the proposed structure dimensions. But the number of resonances is less than the proposed structure . Reference^26^ covers only the Ku band with an EMR of 1.7, much less than the proposed MTM. In Ref.^35^ coverage, the frequency band of X and Ku, respectively, but the EMR value is 1.4, and the dimension of this unit cell is much higher than the proposed structure  . Reference^32^ covers C, X, and Ku bands; even the EMR value is almost similar to the proposed unit cell, but this unit cell did not show the polarization characteristics. Compared to the existing design unit cell in Table 4, the proposed structure  is expressed its its supremacy in size, maximum resonances, covering bands, and independent polarization characterization.

**Table 4:** Comparison between the existing work and proposed work.

Reference	Year	Physical dimension (mm × mm) Electrical dimension (λ × λ)	Operating frequency range (GHz)	Resonance frequency (GHz)	Frequency band	EMR	Polarization-independent
^36^	2021	10.3 × 10.3 0.59 λ × 0.59 λ	5.35–5.69, 17.81–20.67	17.1	Ku	1.7	No
^35^	2018	20 × 20 0.77 λ × 0.77 λ	Not mention	11.5, 13.5	X and Ku	1.4	Yes
^32^	2020	9 × 9 0.125 λ × 0.125 λ	3.95–5.65, 9.57–11.46, 13.68–16.00	4.15, 10.84, 14.93	C, X, and Ku	8.03	No
^21^	2019	5 × 5 0.125 λ × 0.125 λ	6.34–7.39, 8.20–9.98	7.5	C	8	No
^26^	2020	10 × 10 0.12 λ × 0.12 λ	3.42–3.73, 11.27–11.91	3.57, 11.6	S and X	8.4	No
^24^	2018	9 × 8.8 0.125 λ × 0.125 λ	9.655–9.97 10.80–15.00	9.65, 12.6	X and Ku	3.45	No
Proposed	2021	9 × 9 0.125 λ × 0.125 λ	3.70–4.03,7.25–8.27, 10.11–11.37	3.94, 8.08, 11.175	S, C, and X	8.47	Yes

## Conclusion

This study investigated an inverse G-like shape to enable ENG and NZI characteristics for microwave application. The electrical measurement of the structure is 0.118λ × 0.118λ × 0.021 λ, and the EMR value is 8.47, which is calculated at 3.94 GHz. FR-4 is used as a substrate layer, and the resonator is designed on it. The simulated resonance frequencies are 3.94, 8.08 and 11.175 GHz, and the measured result achieves resonance frequencies at 3.96 GHz, 8.18 GHz and 11.20 GHz, including the S, C, and X-bands, respectively. The ENG characteristics exhibited in 3.8–4.17, 7.68–8.54, 10.67–11.36 GHz, respectively. Near-zero refractive index (NZI) is exhibited in 4.07–4.15 and 8.29–8.37 GHz. A widespread investigation has been executed to analyze the E-field and H-field intensity at the resonance frequency. Later, the surface current behavior (circulation and density) is also analyzed to comprehend the structure. This study also manifests the polarization angle insensitivity response of 0°–90° and the incident angle insensitivity response of 0°–60° for TE and TM modes. The CST studio suite 2019 software is performed to extract the effective parameter of the structure. At the lower resonance frequency, the effective medium ratio (EMR) of the MTM unit cell is 8.47. The permittivity and refractive index are shown as the negative characteristics regarded as ENG material. The ENG and NZI properties can be used to enhance the antenna bandwidth and gain and filter designing. The simple design and compactness with high EMR value enable the proposed unit cell for satellite and radar communication. The simulated result agrees with the measured results of the proposed MTM.
